# Specific Gut Microbiome Signatures in Children with Cow’s Milk Allergy

**DOI:** 10.3390/nu16162752

**Published:** 2024-08-18

**Authors:** Dafni Moriki, E. Daniel León, Gabriel García-Gamero, Nuria Jiménez-Hernández, Alejandro Artacho, Xavier Pons, Despoina Koumpagioti, Argirios Dinopoulos, Vassiliki Papaevangelou, Kostas N. Priftis, Konstantinos Douros, M. Pilar Francino

**Affiliations:** 1 3rd Department of Pediatrics, School of Medicine, National and Kapodistrian University of Athens, “Attikon” University Hospital, 12462 Athens, Greece; 2 Department of Genomics and Health, Fundación Para el Fomento de la Investigación Sanitaria y Biomédica de la Comunitat Valencia (FISABIO), 46020 Valencia, Spain; 3 Department of Nursing, National and Kapodistrian University of Athens, 11527 Athens, Greece; 4 CIBER en Epidemiología y Salud Pública, 28029 Madrid, Spain

**Keywords:** gut microbiome, cow’s milk allergy, children, microbial diversity, *Bifidobacterium*, *Clostridium*, hypoallergenic formula

## Abstract

Although gut dysbiosis is associated with cow’s milk allergy (CMA), causality remains uncertain. This study aimed to identify specific bacterial signatures that influence the development and outcome of the disease. We also investigated the effect of hypoallergenic formula (HF) consumption on the gut microbiome of milk-allergic children. 16S rRNA amplicon sequencing was applied to characterize the gut microbiome of 32 milk-allergic children aged 5–12 years and 36 age-matched healthy controls. We showed that the gut microbiome of children with CMA differed significantly from that of healthy children, regardless of whether they consumed cow’s milk. Compared to that of healthy cow’s milk consumers, it was depleted in *Bifidobacterium*, *Coprococcus catus*, *Monoglobus*, and Lachnospiraceae GCA-900066575, while being enriched in *Oscillibacter valericigenes*, *Negativibacillus massiliensis*, and three genera of the Ruminococcaceae family. Of these, only the Ruminococcaceae taxa were also enriched in healthy children not consuming cow’s milk. Furthermore, the gut microbiome of children who developed tolerance and had received an HF was similar to that of healthy children, whereas that of children who had not received an HF was significantly different. Our results demonstrate that specific gut microbiome signatures are associated with CMA, which differ from those of dietary milk elimination. Moreover, HF consumption affects the gut microbiome of children who develop tolerance.

## 1. Introduction

Cow’s milk allergy (CMA) is the most common food allergy in infancy and early childhood. The estimated prevalence in children under three years of age ranges between 0.5 and 3% [1,2]. It usually occurs during the first year of life and may present with various clinical phenotypes, depending on the underlying immune mechanism [3]. In most cases, immune responses are mediated by immunoglobulin E (IgE), but other immune mechanisms (non-IgE-mediated) have also been reported [4]. The clinical course of the disease is favorable, and most children develop tolerance by the age of four years [5,6]. However, in a subgroup of patients, CMA persists into adolescence and adulthood, leading to severe dietary restrictions and thus significantly affecting patients’ health and quality of life [7].

The gut microbiome is a diverse community of bacteria that plays a key role in maintaining homeostasis in the human body. Alterations in the composition of gut microbial populations, referred to as gut dysbiosis, have been associated with the development of various diseases, including food allergies. Emerging evidence suggests that infants with CMA have reduced microbial diversity [8,9] and altered gut microbiome composition compared to healthy controls [8,10,11,12,13,14]. In particular, the gut microbiome of infants with CMA is characterized by increased levels of Proteobacteria and Actinobacteria and a lower abundance of Firmicutes and Bacteroidetes [8,14]. In addition, an increased ratio of Enterobacteriaceae to Bacteroidaceae (E/B ratio) has been observed, as well as depletion of beneficial bacteria such as *Bifidobacterium* and some *Clostridium* species [8,10,11,12,15,16]. It has also been suggested that the gut microbiome composition plays a role in the natural history of the disease [17]. Interestingly, Bunyavanich et al. showed that children with higher levels of Clostridia in the first months of life are more likely to develop tolerance to cow’s milk by the age of 8 years [18].

Although several studies have revealed an association between gut microbiome composition and food allergy, the causal relationship remains unclear. Studies in germ-free mice showed that colonization with certain bacterial strains, such as *Bifidobacterium* and *Clostridium* species, protects against food allergy through direct induction of mucosal regulatory T cells (Tregs) [19,20]. In addition, it has been shown that commensal bacteria regulate the immune system by secreting metabolites such as short-chain fatty acids (SCFAs), which in turn promote the regulatory activity of dendritic cells and lead to the induction of mucosal and peripheral Tregs [21,22,23]. Finally, colonization with certain *Clostridium* species has been shown to improve gut permeability and thus protect against food allergen sensitization and food allergy [24].

Causality is more difficult to establish in humans because of several confounding factors that also affect the composition of the gut microbiome. Undoubtedly, diet is one of them. Especially in the case of CMA, children are deprived of a staple food for a long period, which is likely to have an impact on the gut microbiome. Therefore, it is unclear whether the dysbiosis observed is the cause or the consequence of the disease [25]. Furthermore, children with CMA usually consume hypoallergenic formulas (HFs) as a substitute for cow’s milk, which also seems to have some effect on the gut microbiome, especially when combined with probiotics and prebiotics [11,26,27].

The present study aimed to investigate the taxonomic composition of the gut microbiome of milk-allergic children, to identify taxa that may influence disease expression and the development of oral tolerance. In addition, we aimed to examine the effect of HF consumption on the gut microbiome composition of milk-allergic children.

## 2. Materials and Methods

### 2.1. Study Design

This is a cross-sectional study conducted in collaboration between the Department of Genomics and Health of the Valencian Region Foundation for the Promotion of Health and Biomedical Research (FISABIO) and the Pediatric Allergy and Respiratory Unit of the 3rd Department of Pediatrics of the National and Kapodistrian University of Athens at the “Attikon” University Hospital.

The sampling took place at the Pediatric Allergy and Respiratory Unit of the “Attikon” University Hospital in Athens from January 2021 to December 2023. Due to difficulties in recruiting children, convenience sampling was used. All children with a definite diagnosis of various types of CMA (both IgE-mediated and non-IgE-mediated) were assessed for eligibility. Children aged 5–12 years with a history of CMA (regardless of whether they had developed oral tolerance) and age-matched healthy controls were included in the study. The diagnosis of CMA was based on medical history, detection of specific IgE antibodies against cow’s milk, and oral food challenge tests according to the guidelines of the European Academy of Allergology and Clinical Immunology (EAACI) [28], the American Academy of Allergy, Asthma, and Immunology (AAAAI) [29], and the National Institute for Health and Clinical Excellence (NICE) [30,31].

We excluded (1) children with other food allergies who had not developed oral tolerance, (2) children with chronic gastrointestinal disorders and/or other severe chronic diseases, and (3) children who had received probiotics, corticosteroids, antibiotics, and other medications that affect the gut microbiome three months before enrollment. Age-matched healthy controls were divided according to whether they consumed cow’s milk. In cases of non-milk consumers, a lactose hydrogen breath test was performed according to previous guidelines to exclude children with lactose intolerance [32].

Written informed consent was obtained from the parents of all participants. The study was approved by the Ethics Committee of the University General Hospital “Attikon” (546/1-10-2020).

**Figure 1:**
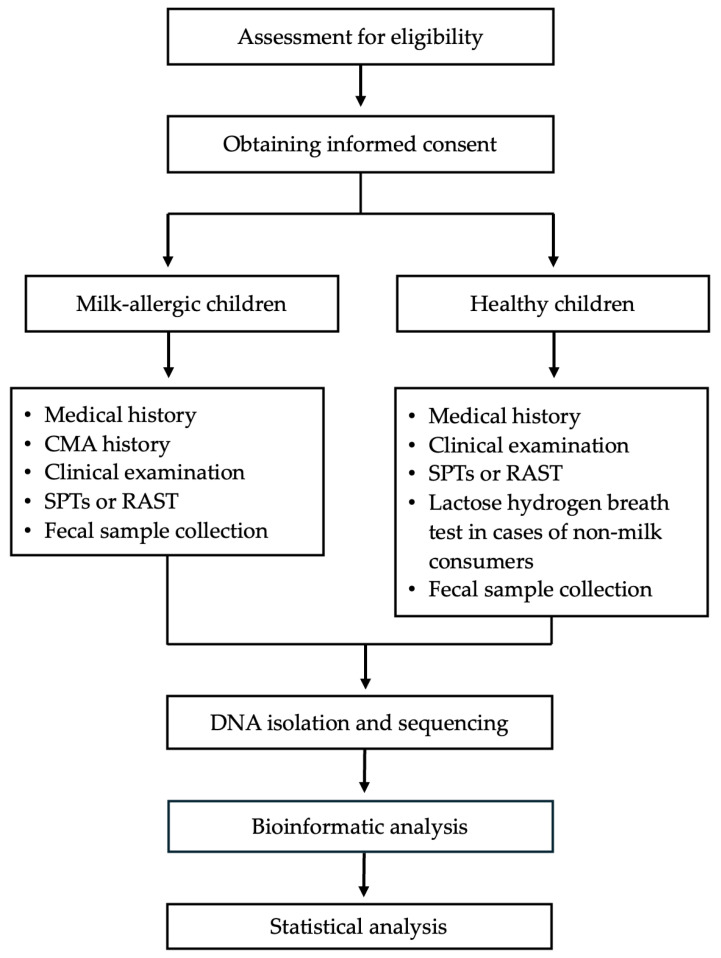
Study design flow chart.

All the steps followed to conduct this study are presented in Figure 1.

### 2.2. Measurements

A detailed medical history was obtained from the parents of all participants at the time of enrollment. Specifically, data on gestational age, mode of delivery, birth weight, type of infant feeding, duration of breastfeeding, number of siblings, age of starting daycare, and family history of allergic diseases were recorded. In children with CMA, additional retrospective data were also collected from parents and medical records on the type of allergy, age at diagnosis, consumption of HF, and age at which oral tolerance developed.

All children were examined by trained pediatricians. Participants’ height and weight were measured to the nearest 0.1 cm and 0.1 kg, after removing outer clothing and shoes. Height and weight were then used to calculate standardized body mass index (BMI) z-score for age and sex using the Centers for Disease Control and Prevention (CDC) growth charts [33]. Participants were also evaluated for other allergic diseases such as atopic dermatitis, allergic rhinoconjunctivitis, and asthma. All children underwent skin prick tests (SPTs) to the most common food allergens (i.e., cow’s milk, casein, whole egg, egg white, yolk, cereals, cod, shrimp, tuna, hazelnut, walnut, almond, peanut, and pistachio) and aeroallergens (i.e., house dust mites, dog and cat dander, olea, cypress, poplar, pine, grasses, artemisia, parietaria, chenopodium, alternaria, and cladosporium). In cases where SPTs could not be performed, blood-specific IgE antibodies (RASTs) were measured. 

### 2.3. Fecal Sample Collection

Fecal samples were collected during study visits or by the parents at home using a stool collection kit provided by the study team. In these cases, samples were stored at 4 °C until they were transported to the research center (within a maximum of 24 h). Then, 10 g of feces was placed in a sterile 50 mL Falcon tube containing 10 mL of RNA later solution and frozen at −80 °C. All samples were sent to FISABIO (Valencia, Spain) in Styrofoam boxes with dry ice.

### 2.4. DNA Isolation and Sequencing

Bacterial pellets obtained from stool samples were lysed using 0.1 mg/mL lysozyme at 37 °C for 30 min. Extraction was performed using the MagNaPure LC JE379 platform and DNA Isolation Kit III (Roche, Mannheim, Germany). Agarose gel electrophoresis (0.8% *w*/*v* agarose in Tris-borate-EDTA buffer) was used to determine DNA integrity. DNA quantification was performed with a Qubit 3.0 Fluorometer (Invitrogen, Waltham, MA, USA), and DNA was then stored at −20 °C until further processing. Following the Illumina protocol for 16S Metagenomic Sequencing Library Preparation, the V3-V4 hypervariable region of the 16S rRNA gene was amplified using 12 ng of DNA. PCR was performed with a forward primer (5′0-TCGT CGGC AGCG TCAG ATGT GTAT AAGA GACA GCCT ACGG GNGG CWGCA-G3′) and reverse primer (5′0-GTCT CGTG GGCT CGGA GATG TGTA TAAG AGAC AGGA CTAC HVGG GTAT CTAA TCC3′0) and linked to adapter sequences for compatibility with the Illumina Nextera XT Index kit. Amplicon libraries were pooled and sequenced in an Illumina Miseq sequencer in 2 × 300 cycles paired-end runs (MiSeq Reagent kit v3, Illumina, San Diego, CA, USA).

### 2.5. Bioinformatic Analysis

The DADA2 (v1.8.0) package in R (v3.6.0) was used for sequence read processing, forward and reverse read merging, and clustering into amplicon sequence variants (ASVs) [34]. Filtering and trimming parameters were as follows: maxN = 0, maxEE = c (2, 5), truncQ = 0, trimLeft = c (17, 21), truncLen = c (270, 220), and rm.phix = TRUE. Specifications for read merging were a minimum overlap of 15 nucleotides and a maximum mismatch of 1 [35]. Taxonomic identification was assigned to ASVs using DADA2 and the SILVA v.138 reference database.

### 2.6. Statistical Analysis

Statistical analysis of demographic and clinical data was performed using SPSS 29 software (IBM SPSS Statistics, version 29.0.1.1). Continuous variables are presented as mean values with standard deviation (SD). Categorical variables are expressed using absolute and relative frequencies. Differences between groups were assessed using Pearson’s chi-square test for categorical variables and ANOVA or Kruskal–Wallis test for continuous variables, as appropriate. All reported probability values (*p*-values) were compared at a significance level of 5%.

Beta diversity analyses were performed with the vegan package (v2.5-2) on the R platform. The Bray–Curtis dissimilarity index was employed to obtain the general measure of dissimilarity between two microbial communities and was used in the permutational multivariate analysis of variance (PERMANOVA). The adonis function included in the Vegan package with 600 permutations was applied to perform PERMANOVA. Analysis of the composition of microbiomes (ANCOM) was used to identify the presence of differentially abundant taxa among samples. A normalized table was obtained from ANCOM, then we converted the ANCOM matrix to positive data by adding the smallest value to the entire matrix. Once all the data were positive, we performed the Wilcoxon rank-sum test to evaluate the significance of abundance differences. The Benjamin–Hochberg procedure was used for false discovery rate control as described in Kaul et al. [36].

## 3. Results

### 3.1. Clinical and Demographic Characteristics

Sixty-eight children (32 milk-allergic and 36 healthy controls), aged 5–12 years, were included in the study. This sample size is in line with those of previous case–control studies that have detected taxon abundance differences in the gut microbiome of cow’s milk-allergic and healthy children based on 16S rRNA gene sequencing [37]. The mean age of the children was 7.3 (2.1) (standard deviation, (SD)) years, and 35 (51.5%) were boys. Milk-allergic children were further divided according to whether they had developed oral tolerance to cow’s milk (DOT) or not (CMA). Age-matched healthy controls were divided according to whether they consumed cow’s milk (H) or not (HNMC). Clinical and demographic characteristics of all children are presented in Table 1.

Among the allergic children, 18 had IgE-mediated CMA, and 14 had non-IgE-mediated CMA. Other clinical characteristics of the allergic children are presented in Table 2.

### 3.2. Gut Microbiome Composition in Milk-Allergic and Healthy Children

**Figure 2:**
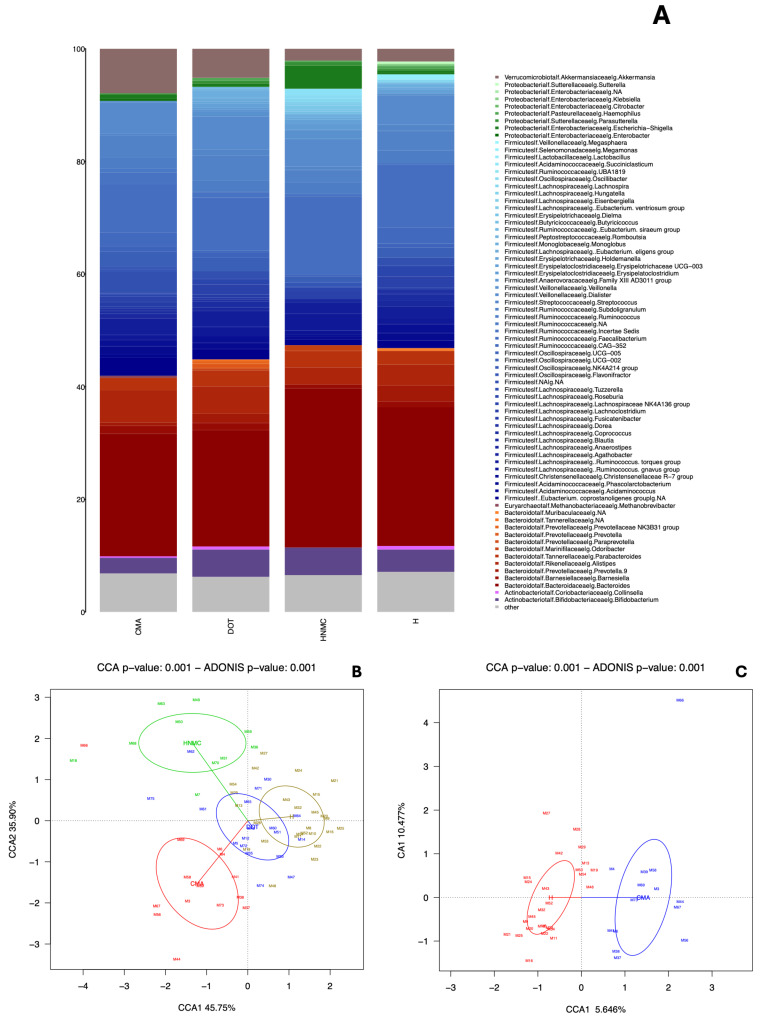

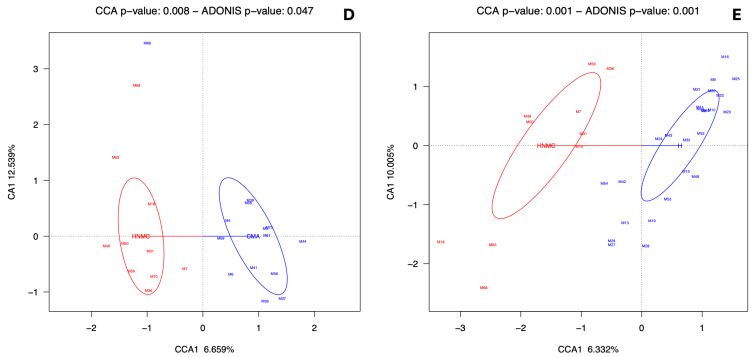
Differences in gut microbiome composition. (**A**) The relative abundance of genera across groups is represented using a bar plot. (**B**) Canonical correlation analysis (CCA) of H, HNMC, CMA, and DOT shows significant differences in microbiome composition. The DOT and H groups showed similar microbiome compositions, whereas the CMA and HNMC groups were not identical to the H and DOT groups, nor each other. Permutational multivariate analysis of variance (PERMANOVA) was conducted to determine whether there were significant differences between the groups (**C**) H vs. CMA, (**D**) HNMC vs. CMA, and (**E**) HNMC vs. H.

We first compared the overall composition of the gut microbiome of the children in the four groups (i.e., H, HNMC, DOT, and CMA) (Figure 2A,B). PERMANOVA analysis confirmed that there were statistically significant differences between them (Figure 2B). Pairwise comparisons also revealed statistically significant differences. Specifically, children with CMA had a significantly different gut microbiome composition compared to healthy children, regardless of whether they consumed cow’s milk (Figure 2C) or not (Figure 2D). In contrast, the overall gut microbiome composition of children who developed tolerance (DOT) did not differ significantly from that of children with CMA, nor from that of healthy children (H and HNMC). Finally, the gut microbiome of healthy children differed significantly depending on whether they consumed cow’s milk or not (Figure 2E).

Differential abundance analyses revealed that the gut microbiome of children with CMA was depleted in *Bifidobacterium*, *Coprococcus catus*, *Monoglobus*, and Lachnospiraceae GCA-900066575 in comparison to that of healthy children consuming cow’s milk (H), while being enriched in *Oscillibacter valericigenes*, *Negativibacillus massiliensis*, Ruminococcaceae *incertae sedis*, and two unclassified genera of the Ruminococcaceae family. Interestingly, although a statistically significant difference was observed between the overall gut microbiome composition of children with CMA and healthy controls who did not consume cow’s milk (HNMC) (Figure 2D), differential abundance analyses revealed no statistically significant differences between the two groups for any single bacterium. On the other hand, *Eisenbergiella massiliensis*, Ruminococcaceae *incertae sedis*, *Parasutterella excrementihominis*, *Porphyromonas*, *Ruminococcus bromii*, *Acetanaerobacterium*, and unclassified Ruminococcaceae were more abundant in HNMC compared to H.

### 3.3. Effect of HF Consumption on the Gut Microbiome Composition in Milk-Allergic Children

**Figure 3:**
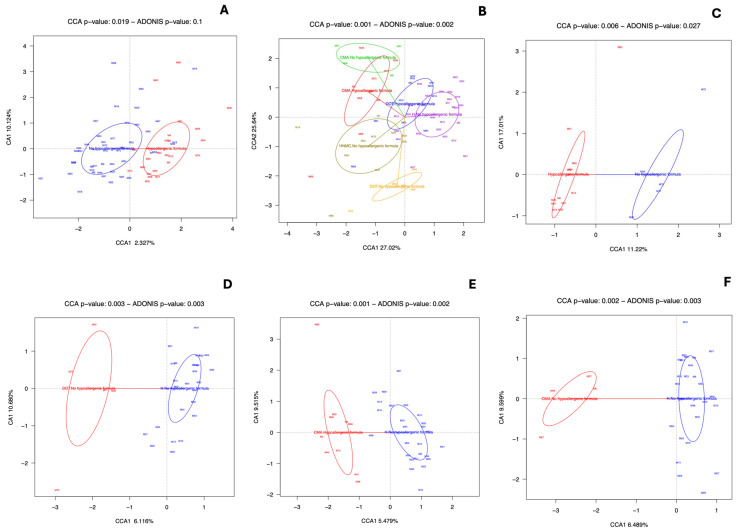
Effect of HF consumption on gut microbiome composition. (**A**) CCA analysis showed no statistical difference between all children fed with HF and all children not fed with HF. (**B**) CCA showed statistical variation among groups. Interestingly, for the CMA group, HF consumption had no significant impact on bacterial composition. In contrast, in the DOT group, it had a beneficial effect: those fed with HF had a gut microbiome composition more similar to that of healthy children. Pairwise comparison showed statistically significant differences between (**C**) DOT children fed with HF vs. DOT children not fed with HF, (**D**) DOT children not fed with HF vs. healthy children (not fed with HF), (**E**) children with CMA fed with HF vs. healthy children (not fed with HF), and (**F**) children with CMA not fed with HF vs healthy children (not fed with HF).

To examine the effect of HF consumption on the gut microbiome composition, we compared children of all categories who were fed with an HF with those who were not. No statistically significant correlations were found (Figure 3A). However, when we divided the two groups of milk-allergic children (CMA and DOT) according to whether they had consumed an HF, statistically significant differences were observed (Figure 3B). In particular, it was shown that the gut microbiome of children who developed tolerance (DOT) and had consumed an HF as a substitute was similar to that of healthy children. In contrast, DOT children who had not consumed an HF had the least similar gut microbiome to all groups. 

Pairwise comparison showed that children who developed tolerance (DOT) had a significantly different bacterial composition depending on whether they were fed with an HF (Figure 3C). However, no statistically significant differences were observed in any specific bacterial taxa. Furthermore, the gut microbiome of children who developed tolerance (DOT) and had not consumed an HF differed significantly from that of the healthy controls (Figure 3D) and was enriched in *Veillonella*, Ruminococcaceae *incertae sedis*, *Veillonella dispar*, *Bifidobacterium longum*, and Lachnospiraceae *(Eubacterium) fissicatena group.* Statistically significant differences were also observed between the composition of the gut microbiome of healthy children and children with CMA, regardless of whether they were fed with an HF (Figure 3E) or not (Figure 3F). Nevertheless, no statistically significant differences in specific bacteria were observed.

## 4. Discussion

In the present study, we sought to investigate the taxonomic composition of the gut microbiome of milk-allergic children and to examine the effect of HF consumption on it. We also showed that cow’s milk consumption has a significant effect on the gut microbiome of healthy children. Nevertheless, we showed that the gut microbiome composition of children with CMA differs significantly from that of healthy children, regardless of whether they consume cow’s milk. Therefore, we can conclude that the dysbiosis observed in children with CMA is not only a signature of the milk elimination diet but also a signature of the disease. In addition, we showed that the gut microbiome of milk-allergic children who developed tolerance did not differ significantly from that of children who still had CMA. This implies that the structure of the gut microbiome of milk-allergic children is not fully restored despite the development of oral tolerance. However, this should be interpreted with caution, as the lack of a statistically significant difference may be due to the small number of children included. Finally, we showed that HF consumption has a significant impact on the composition of the gut microbiome in children who develop tolerance, making the microbiome of those who consumed it similar to that of healthy children. 

Several studies have shown that the gut microbiome of children with CMA differs significantly from that of healthy children and this has been suggested to play a role in the development of the disease [8,9,10,11,13,38,39]. However, the causal relationship has not been established [25]. To determine whether the dysbiosis observed could simply be the consequence of the lack of milk consumption associated with the disease, we analyzed the gut microbiome of children with CMA and compared it with that of healthy children who consumed cow’s milk and those who did not. Our findings support the hypothesis that the dysbiosis observed is not only due to milk elimination but is also specifically associated with the disease. Nevertheless, it is not yet known which specific bacterial taxa are responsible and by which underlying mechanism they may contribute to the disease. So far, the depletion of certain beneficial bacteria, such as *Bifidobacterium* and some *Clostridium* species [8,10,11,12,15,16], as well as the increase in *Alistipes* and other Bacteroidetes [38] have been implicated. 

In this cross-sectional study, we identified nine genera from the Ruminococcaeceae, Bifidobacteriaceae, Lachnospiraceae, Oscillospiraceae, and Monoglobaceae families that were differentially abundant in children with CMA compared to healthy controls that consumed cow’s milk. Consistent with other studies [37], we found that *Bifidobacterium* was depleted in children with CMA. This genus, which is usually abundant in the gut of breastfed infants, possesses a unique fructose-6-phosphate phosphoketolase pathway that is used to ferment carbohydrates to produce lactate, acetate, and ethanol [40]. Lactate accumulation has the potential to significantly alter the intestinal microenvironment and, consequently, change the gut microbiome composition by reducing pH levels [41]. Therefore, lactate can inhibit the growth of certain bacterial pathogens with poor tolerance to low pH such as *Escherichia coli* [42]. In addition, *Bifidobacterium* has been shown to suppress skewed T helper (Th) 2 immune responses in allergic mice and induce Foxp3^+^ Tregs. Therefore, it improves the impaired function of the intestinal epithelial barrier and promotes oral tolerance to food antigens [19,43]. The gut microbiome of children with CMA was also depleted in *Coprococcus catus* and Lachnospiraceae GCA-900066575. These bacteria are members of the Lachnospiraceae family of the Clostridia class, which is involved in the fermentation of non-digestible polysaccharides into SCFAs [44]. In particular, *Coprococcus catus* utilizes lactate to produce butyrate and propionate. These metabolites are potent anti-inflammatory mediators, enhance the regulatory function of dendritic cells, and lead to the induction of mucosal and peripheral Tregs [21,22,23]. In addition, experimental research has shown that several members of the Lachnospiraceae protect against food allergy through stimulating the production of IL-22 which reinforces the epithelial barrier to reduce gut permeability to dietary proteins [24]. Interestingly, an enrichment of bacteria from the Clostridia class has been observed in the gut of infants who outgrew CMA by the age of 8 years [18]. Furthermore, since excessive lactate production has been linked to harmful effects on human health, it has been suggested that *Coprococcus catus* exerts an indirect beneficial effect through the consumption of this metabolite [45]. The gut microbiome of children with CMA also contained a reduced relative abundance of *Monoglobus.* The latter is a pectin-degrading bacterium that has been negatively associated with intestinal inflammation [46]. *Monoglobus* has recently been shown to produce bile acid metabolites that regulate Th17 cells, which are critical for barrier integrity [47]. Specifically, *Monoglobus* converts the secondary bile acid lithocholic acid (LCA) to 3-oxoLCA and iso-alloLCA, which in turn inhibit the differentiation of Th17 cells and increase the differentiation of Tregs. Thus, these metabolites enhance the integrity of the intestinal barrier and possibly through this mechanism protect against food allergy. 

On the contrary, the gut microbiome of children with CMA was enriched in *Oscillibacter valericigenes*, *Negativibacillus massiliensis*, Ruminococcaceae *incertae sedis*, and two other unclassified genera of the Ruminococcaceae family. *Oscillibacter valericigenes* belongs to the Clostridia class and is a harmful bacterium associated with inflammation and insulin resistance. This bacterium has been shown to increase metabolically damaging macrophages in adipose tissue through the production of Toll-like receptor (TLR) agonists [48]. In addition, *Oscillibacter valericigenes* produces valeric acid, an SCFA that has been reported to have an inhibitory effect on histone deacetylase isoforms (HDAC) [49]. Recently, Kourosh et al. showed that *Oscillibacter valericigenes* was increased in the gut of children with IgE-mediated food allergy, especially in children older than 7 years of age, compared to their healthy siblings and age-matched healthy controls [50]. However, the exact function of this bacterium and how it is linked to food allergy remains unknown. *Negativibacillus massiliensis* is a newly classified bacterium that has been associated with diamine oxidase (DAO) levels, a biomarker of intestinal barrier damage [51]. This enzyme regulates the degradation of histamine in rapidly proliferating tissues such as the intestinal mucosa [52]. To our knowledge, this is the first time that *Negativibacillus massiliensis* has been linked to food allergy. We also showed that three taxa of the Ruminococcaceae family were more abundant in children with CMA. Previous studies have shown that the members of this family, which also belongs to the Clostridia class, are overrepresented in the gut of children with food allergies [11,53]. Interestingly, a recent prospective study showed that the Ruminococcaceae family was underrepresented in 6-month-old infants who developed allergic diseases, but then increased in allergic compared to non-allergic children in a time-dependent manner [54]. It is therefore likely that not all members of the Clostridia class are beneficial [55]. In addition, previous studies have reported an increased abundance of the genera *Ruminococcus* and *Subdoligranulum* as well as *Clostridium coccoides* and *Clostridium celerecrescens* in the gut of infants with CMA [11,13,16,26]. In contrast to other studies, we found no significant differences in any taxa of the phylum Bacteroidetes, including *Bacteroides* [16], *Alistipes* [38], and *Prevotella* [9].

For comparison, we also examined the effect of a milk-elimination diet on the gut microbiome composition of healthy children. We identified seven taxa that differentiate children who consumed cow’s milk from those who did not. The majority of these taxa belong to the Clostridia class. Interestingly, no statistically significant differences were observed in the lactose-fermenting *Bifidobacterium*. Furthermore, *Ruminococcus bromii*, which ferments lactose and produces butyrate, was more abundant in non-milk consumers. This contrasts with the results of previous studies showing that in vitro fermentation of dairy products leads to a higher abundance of *Ruminococcus bromii* [35]. However, this bacterium is involved in the degradation of resistant starch and the consumption of other starch-rich foods may have affected the results [56]. Importantly, the taxa detected to have differential abundances between healthy milk consumers and healthy non-milk consumers mostly differed from those detected in the comparison between healthy milk consumers and CMA children. In fact, only Ruminococcaceae *incertae sedis* and other unclassified Ruminococcaceae were detected in both comparisons, supporting the fact that milk elimination alone cannot explain the microbiome composition of CMA children and that other differences between CMA children and healthy milk consumers should be specifically attributed to the disease itself. 

On the other hand, although we found a statistically significant difference when comparing the overall microbiome composition between children with CMA and healthy controls who did not consume cow’s milk, we could not identify specific bacterial taxa that were differentially abundant between the two groups, possibly due to the low number of children in the latter category. Similarly, although there is evidence that specific gut microbiome signatures are associated with the acquisition of immune tolerance to food antigens [15,18], we were unable to identify specific bacterial taxa that may influence this process. We showed that the gut microbiome composition of children who developed tolerance was not significantly different from that of children who still had CMA, but that it no longer differed significantly from that of healthy children. This suggests that their microbiome displays an intermediate state and that studies involving larger population sizes or children who have been tolerant for longer spans of time may detect a shift from the microbiome composition associated with CMA. 

Currently, there is no effective treatment for CMA other than complete avoidance of milk and dairy products and emergency medical treatment in cases of unintentional exposure. Therefore, since milk is a primary food in infancy and early childhood, most children receive an HF as a substitute. In the present study, we sought to investigate whether HF consumption has an effect on the gut microbiome composition of milk-allergic children. Although our findings support the idea that HF consumption affects the composition of the gut microbiome of milk-allergic children who develop tolerance and makes it similar to that of healthy children, we were unable to identify specific bacterial taxa associated with it. As has already been said, this is probably related to the small sample size of the present study. In addition, further analyses with different omic technologies are required to determine whether functional changes also accompany the observed changes in composition.

The primary objective of our study was to investigate and characterize the gut microbiome of children with CMA and healthy controls. The comparisons made indicate key differences between the two groups. Another contribution of our work is the observation that children with CMA who develop tolerance have a gut microbiome composition that is similar to that of healthy children if they have received an HF as a substitute. Once these differences are further characterized in larger populations and we are able to understand the mechanism by which specific bacteria contribute to food allergy, we can use this knowledge to design new therapeutic approaches to prevent and control the severity of symptoms or even treat the disease.

The present study has several limitations. First of all, it has a cross-sectional design and hence suffers from the inherent limitations of this type of observational study, including the inability to draw causal inferences and recall bias. The use of a convenience sample also undermines the generalizability of the results. In addition, the small sample size and the fact that it included children with both IgE-mediated and non-IgE-mediated CMA probably precluded the detection of more statistically significant associations. Furthermore, we did not examine the effect of dietary factors other than milk consumption on gut microbiome composition.

## 5. Conclusions

In conclusion, in this study, we showed that children with CMA display specific microbial signatures associated not only with the milk elimination diet but also with the disease itself. This implies that certain bacterial populations are essential for maintaining gut homeostasis and contribute to the development of oral tolerance. Therefore, identifying these bacteria and understanding the underlying mechanisms that lead to immune tolerance may be the key to preventing and treating the disease. Furthermore, we demonstrated that the consumption of an HF as a substitute has a significant effect on the gut microbiome of milk-allergic children. In particular, children who develop tolerance have a gut microbiome composition similar to that of healthy children if they have been fed with an HF. This suggests that the consumption of an HF as a substitute may be important for children with CMA. Nevertheless, further research is needed to confirm this and to determine whether functional changes also accompany the observed changes in composition. In addition, larger prospective cohort studies and clinical trials are needed to better understand the role of the gut microbiome and explore its therapeutic potential in children with food allergies.

## Figures and Tables

**Table 1:** Main demographic and clinical characteristics of the study population (*n* = 68).

	Healthy Controls		Milk-Allergic Children		
Characteristics	Consumed Cow’s Milk		Developed Oral Tolerance		*p*
	Yes (H)	No (HNMC)	Yes (DOT)	No (CMA)	
*n*	26	10	18	14	
Male, *n* (%)	14 (53.8)	5 (50)	9 (50)	7 (50)	0.99
Age (years), mean (SD)	7.4 (2.0)	7.9 (2.3)	7.3 (2.2)	6.7 (2.0)	0.49
BMI (z-score), mean (SD)	0.38 (0.90)	0.51 (1.08)	−0.03 (1.41)	−0.32 (1.29)	0.26
Gestational age (weeks), mean (SD)	38 (37–39)	37.5 (34–38)	38 (37–40)	38 (38–39)	0.07
Vaginal delivery, *n* (%)	11 (42.3)	1 (10)	7 (38.9)	6 (42.9)	0.29
Birth weight (g), mean (SD)	3064 (555)	2672 (729)	2932 (471)	3199 (350)	0.17
Type of infant feeding *n* (%)					
Breastfeeding	14 (53.8)	5 (50)	9 (50)	9 (64.3)	0.77
Formula feeding	2 (7.7)	2 (20)	4 (22.2)	1 (7.1)	
Mixed feeding	10 (38.5)	3 (30)	5 (27.8)	4 (28.6)	
Having an older sibling, *n* (%)	13 (50)	5 (50)	7 (38.9)	6 (42.9)	0.89
Age of starting daycare (years), mean (SD)	3.1 (1.1)	3.0 (1.0)	2.6 (1.1)	3.0 (0.9)	0.5
Atopic history, *n* (%) *	8 (30.8)	5 (50)	14 (77.8)	10 (71.4)	0.009
Family history of allergic diseases, *n* (%)	15 (57.7)	7 (70)	16 (88.9)	12 (85.7)	0.08

H: healthy children; HNMC: healthy non-milk consumers; DOT: developed oral tolerance; CMA: cow’s milk allergy; BMI: body mass index; * History of allergic disease other than food allergy (i.e., atopic dermatitis, allergic rhinitis or rhinoconjunctivitis, and asthma).

**Table 2:** Clinical characteristics of milk-allergic children.

	Developed Oral Tolerance		
Characteristics	Yes (DOT)	No (CMA)	*p*
*n*	18	14	
Type of allergy, *n* (%)			
IgE-mediated	7 (38.9)	11 (78.6)	
Allergic proctocolitis	7 (38.9)	0 (0)	
FPIES	0 (0)	2 (14.3)	
Other non-IgE-mediated	4 (22.2)	1 (7.1)	
Age at diagnosis (months), mean (SD)	4.1 (4.2)	6.1 (5.7)	0.12
Fed with HF, *n* (%)	13 (72.2)	10 (71.4)	0.96
Duration of HF consumption (years), mean (SD)	1.6 (1.4)	4.8 (2.4)	<0.001
Duration of breastfeeding (months), mean (SD)	15.0 (15.4)	16.2 (17.4)	1.00

DOT: developed oral tolerance; CMA: cow’s milk allergy; IgE: immunoglobulin E; FPIES: food protein induced enterocolitis syndrome; HF: hypoallergenic formula.

## Data Availability

The 16S rRNA gene sequences employed in this study can be found at https://www.ebi.ac.uk/ena, accessed on 30 July 2024, under accession number PRJEB78556. Other datasets are available on request from the authors.
